# Tunable Magnetization Dynamics in Interfacially Modified Ni_81_Fe_19_/Pt Bilayer Thin Film Microstructures

**DOI:** 10.1038/srep17596

**Published:** 2015-12-01

**Authors:** Arnab Ganguly, Sinan Azzawi, Susmita Saha, J. A. King, R. M. Rowan-Robinson, A. T. Hindmarch, Jaivardhan Sinha, Del Atkinson, Anjan Barman

**Affiliations:** 1 Department of Condensed Matter Physics and Material Sciences, S. N. Bose National Centre for Basic Sciences, Block JD, Sec. III, Salt Lake, Kolkata 700098, India; 2 Department of Physics, Durham University, Durham, DH1 3LE, United Kingdom

## Abstract

Interface modification for control of ultrafast magnetic properties using low-dose focused ion beam irradiation is demonstrated for bilayers of two technologically important materials: Ni_81_Fe_19_ and Pt. Magnetization dynamics were studied using an all-optical time-resolved magneto-optical Kerr microscopy method. Magnetization relaxation, precession, damping and the spatial coherence of magnetization dynamics were studied. Magnetization precession was fitted with a single-mode damped sinusoid to extract the Gilbert damping parameter. A systematic study of the damping parameter and frequency as a function of irradiation dose varying from 0 to 3.3 pC/μm^2^ shows a complex dependence upon ion beam dose. This is interpreted in terms of both intrinsic effects and extrinsic two-magnon scattering effects resulting from the expansion of the interfacial region and the creation of a compositionally graded alloy. The results suggest a new direction for the control of precessional magnetization dynamics, and open the opportunity to optimize high-speed magnetic devices.

Controlling the magnetic properties of micro- and nano-scale magnetic structures is important for applications, such as magnetic data storage and sensors, and emerging technology concepts in spintronics^1^ and magnonics^2^,^3^. This drives the search for engineered magnetic materials and metamaterials. In general ferromagnetic (FM) materials with low Gilbert damping are preferred in spin-transfer torque magnetic random access memory and magnonic devices, for reduced write-current and enhanced propagation of spin waves, respectively. On the other hand, for data storage and memory devices higher damping aids suppression of the magnetization precession during writing. Currently, to achieve control over spin waves^4^ lithographic patterning of magnonic crystals^5^,^6^,^7^,^8^ was introduced, while for magnetic domain wall devices the behavior has been controlled by the lithographically defined shape^9^ of nanowire structures and anisotropy^10^ has been introduced by patterning magnetic films. Such patterning requires complicated nanofabrication methods, which, on a smaller scale may introduce defects that significantly affect the magnetic behaviour. Investigations of magnetic bilayers and multilayers have revealed interesting phenomena like spin-dependent scattering^11^ in giant magnetoresistance (GMR) multilayers, interlayer exchange coupling, interface hybridization, spin injection^12^,^13^ and spin pumping^14^, where the interface plays a crucial role in controlling the magnetization dynamics. Hence, interfacial engineering in ferromagnetic systems offers an exciting opportunity to both explore fundamental aspects of the interface physics and address the technological requirements for control of magnetization behavior as an alternative or complement to control by patterning. Precessional magnetization dynamics represents the transient response of the magnetization triggered by an external stimulus, such as a magnetic field pulse or a laser pulse. The magnetization vector follows a damped oscillatory trajectory in time-domain governed by the internal magnetization relaxation processes and the external magnetic field (*H*). The dynamical motion is phenomenologically described by Landau–Lifshitz–Gilbert (LLG) equation^15^


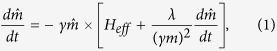


where, 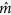 is the magnetization, 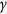 is the gyromagnetic ratio, *H*_*eff*_ is the effective magnetic field and 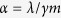 is the Gilbert damping coefficient. The relaxation of magnetization dynamics is governed by the damping parameter, *α*, which describes how rapidly magnetization equilibrates in absence of any stimulus. As introduced earlier, *α* plays an important role in technology, including magnetization switching in spin valves^16^, spin-injection and detection^17^, current-induced magnetization reversal^16^, and spin wave propagation in magnetic media^18^. Despite significant research efforts and industrial need, the nature and origin of all the contributing factors to damping are not fully understood. A combination of spin-orbit interactions^19^ and *s-d* scattering into ferromagnetic band electrons^20^ provides some understanding of the origin of magnetic relaxation giving rise to intrinsic mechanisms for damping, while, extrinsic contributions, in particular the role of defects, via so-called two-magnon scattering^21^, have also been shown to enhance *α* and leads to a shift in the precessional frequency, *f*, for in-plane magnetized thin ferromagnetic films. This mechanism may be visualized by the generation of higher frequency spin waves that are degenerate with the FMR mode in the presence of dipolar coupling between spins. The defects scatter energy from the FMR mode into the spin waves leading to a relaxation with some de-phasing. In the case of bilayered or multilayered thin films the situation is further complicated because the intrinsic damping in the system is sensitive to both electronic hybridization at the interface and spin pumping into non-magnetic (NM) layers. As outlined, a number of theoretical models exist for explaining damping analytically, however, experimental assessment is challenging, as it is difficult to distinguish the effects contributing to the experimentally observed damping.

In this work the role of interfacial structure on the damping of Ni_81_Fe_19_(NiFe)/Pt bilayer films was studied by engineering the interface via exposure to low dose Ga^+^ ion irradiation using a focused ion beam (FIB) system. The low dose regime principally leads to interfacial intermixing via ballistic cascade along with low levels of gallium implantation (up to ~1%) and limited sputter loss from surface of the bilayer. Recently, FIB has been used to locally manipulate magnetic properties like precession frequency and damping^22^ by controlled intermixing with low-dose ion irradiation. The variation of the effective damping and the precession frequency, and importantly, the spatial coherence of the dynamics were determined using a time-resolved magneto-optical Kerr effect microscope (TR-MOKE).

## Results and Discussion

**Figure 1:**
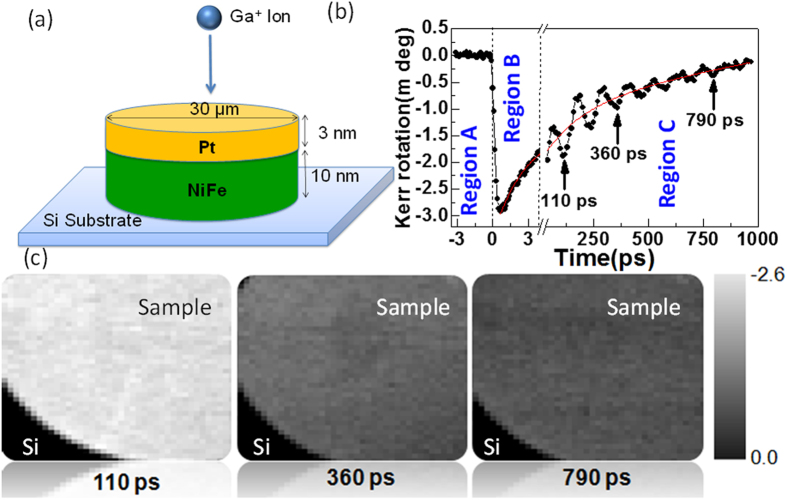
(**a**) Schematic illustration of ion beam irradiation of a bilayer circular structure. (**b**) TR-MOKE trace from a sample irradiated with ion dose d = 3.1 pC/μm^2^. Note changes in the time base. (**c**) Time-resolved Kerr images of the sample at three time delays.

A bilayer of NiFe(10 nm)/Pt(3 nm) was grown by sputtering on a thermally oxidised Si[100] substrate using a ultrahigh vacuum deposition system from a base pressure ~1 × 10^−8^ Torr. The bilayer films were patterned by electron beam lithography into 30 μm disks and were irradiated with irradiation dose (d) varying from 0 to 3.3 pC/μm^2^. Figure 1(a) shows a schematic diagram of the sample. NiFe was chosen for its negligible anisotropies. Pt was selected as an important heavy metal with regard to its strong spin-orbit coupling^23^ and significant proximity induced magnetic moment, although the role of this is currently an open question. Structurally, the as-deposited NiFe/Pt interface is shown to have a typical width of less than 1 nm, resulting form a combination of topological roughness and chemical intermixing as obsrved here by x-ray reflectivity. It has been shown in a previous detailed structural study of NiFe/Au that with higher ion dose the interface rapidly becomes broader, the capping layer becomes very thin and the layer develops into a compositionally-graded alloy due to the intermixing of atoms of the heavy NM layer into the FM layer^24^,^25^. Pt also forms a protective layer preventing the oxidation of the NiFe.

Time-resolved magnetization dynamics of the samples were measured using a custom built TR-MOKE microscope. An in-plane bias magnetic field (*H*) was applied at an angle of ~5° to the plane of the sample. The magnetization 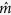 was aligned uniformly along the direction of *H*. An all-optical femtosecond laser-based pump-probe method was used to excite the magnetization dynamics and to probe the evolution of magnetization as a function of delay time with respect to the excitation. Details of the setup are described elsewhere^26^. Kerr images of the 30 μm disks were built-up by raster scanning the sample under the probe spot using an x-y-z piezoelectric scanning stage at fixed time delays and by measuring the Kerr rotation at each scan point. The experiments were performed at room temperature.

Figure 1(b) shows typical time-resolved Kerr rotation data obtained from a bilayer disk irradiated with d = 3.1 pC/μm^2^ measured under *H* = 0.8 kOe. Three different temporal regimes are identified. In region A (t < 0) a negative time delay simply shows steady magnetization signal due to *H* when the sample is probed prior to excitation. A change in the Kerr angle from this state shows a change in the magnetization from its initial uniform state. In region B ultrafast demagnetization occurs within about 500 fs as a result of the pulsed laser excitation, the Kerr signal recovers quickly (relaxation time 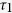) as the electronic thermal bath equilibrates with the lattice. This is followed by a slower relaxation with time constant 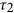 in region C where the lattice dissipates energy to the surrounding^27^. The solid curves are exponential fits to the relaxation in regions B and C. These relaxation processes are governed by the specific heats and the coupling between the different energy systems. A damped oscillatory signal was also observed in region C superimposed upon the decaying signal, and is due to the precession of magnetization that is described by Equation 1.

### Time-resolved scanning Kerr images

Figure 1(c) shows scanning Kerr images^28^ of the same region of one of the irradiated disks at three different antinodes of the precession (time delays = 110 ps, 360 ps, 790 ps as indicated by the arrows in Fig. 1(b)). These images show the uniformity of the excitation within the circular structures and decreasing brightness (Kerr angle) with increasing time delay as the precession is damped. This indicates that any significant contribution to the damping from dephasing of multiple spin wave modes is ruled out and the damping is spatially uniform. Similar uniformity was observed for samples with other doses. This confirms that the magnetic changes due to irradiation were uniform over the sample and independent of the region probed. The effect of finite boundary or demagnetized region can also be excluded here since the sample size is much larger than the excitation and probed regions of about 1μm^2^.

### Precession frequency and damping

**Figure 2:**
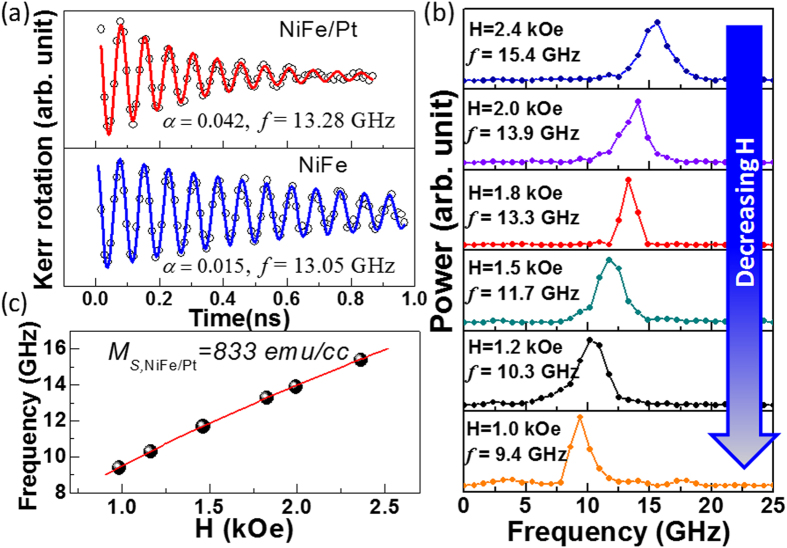
(**a**) Comparison of TR-MOKE traces between NiFe/Pt (d = 0.3 pC/μm^2^) and NiFe films. Symbols correspond to experimental data while the solid curves are fits to Equation 2. (**b**) Power spectra of NiFe/Pt sample at different bias field values *H*. (**c**) Frequency vs. bias magnetic field for the NiFe/Pt (d = 0.3 pC/μm^2^) sample. Here the symbols represent experimental data points and the solid curve is a fit to Equation 3.

A bi-exponential background was subtracted from the TR-MOKE traces to isolate the damped precessional behavior. Precessional data for a bilayer structure irradiated with d = 0.3 pC/μm^2^ and uncapped NiFe film at *H* = 1.8 kOe are presented in Fig. 2(a). In both cases a single frequency damped oscillation is clear and the decay is much faster for the bilayer film. Similar single frequency damped oscillatory behaviour was obtained for all of the samples here, see Fig. 1(c) for further examples. The time domain data were fitted with a damped sine curve:


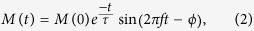


where the relaxation time 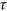 is related to the damping coefficient *α* by the relation 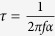, *f* is the precession frequency and *ϕ* is the initial phase of the oscillation. Examples of best fits to the experimental data are shown by the solid curves in Fig. 2(a), from which *α* values of 0.042 for the NiFe/Pt bilayer and 0.015 for the uncapped NiFe layer were extracted. The uncapped value is consistent with typical values for NiFe film and the Pt capping increased the damping by almost a factor of 3. This large increase in damping may be attributed to spin pumping^29^,^30^ into the Pt and enhanced dissipation associated with increased spin-orbit coupling (SOC)^23^ at the NiFe/Pt interface. The precession frequency for NiFe/Pt (13.28 GHz) was slightly higher than for the uncapped NiFe (13.05 GHz), which may perhaps be attributed to an induced moment on the interfacial Pt atoms. Figure 2(b) shows a monotonic decrease of *f* with decreasing *H* for the NiFe/Pt system from the fast Fourier transform (FFT) power spectra and Fig. 2(c) shows *f* plotted as a function of *H*, and fitted with the Kittel formula for uniform precession, from which the saturation magnetization *M*_*S*_ was obtained as 833 emu/cc with the gyromagnetic ratio 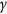 = 0.0176 GHz/Oe.


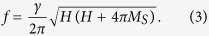


**Figure 3:**
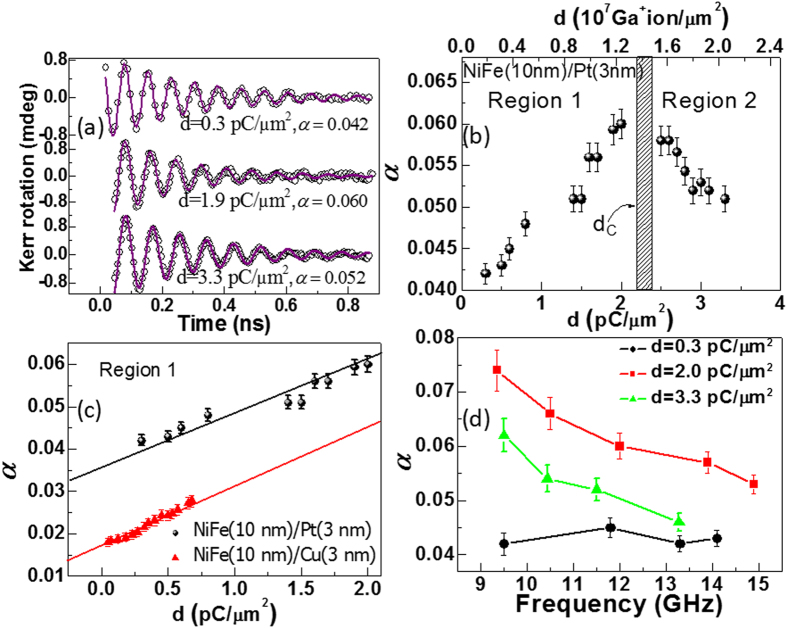
(**a**) TR-MOKE traces at three different doses. Here, symbols correspond to the experimental data and solid curves are fits to Equation 2. (**b**) Damping is plotted as a function of dose. The shaded box represents the transion between two regions. (**c**) Variation of damping with dose in the lower dose regime for NiFe/Pt (filled circles) and NiFe/Cu (filled triangles). Here, symbols are the experimental results and solid lines are linear fits. The data shown in Fig. 3 (a–c) correspond to *H* = 1.8 kOe. (**d**) Variation of damping with precession frequency at three different doses.

The influence of interfacial engineering on the precession and damping of NiFe/Pt bilayers is shown in Fig. 3(a). For the lowest irradiation dose (d = 0.3 pC/μm^2^) a value of *α* = 0.042 was obtained, while for an intermediate dose, d = 2.0 pC/μm^2^, *α* increased to a maximum of 0.060 before falling again to *α* = 0.052 for the largest dose, d = 3.3 pC/μm^2^.

In Fig. 3(b) the dependence of damping on the ion beam dose is plotted, where the top *x*-axis shows the number density of Ga^+^ ions used during irradiation. The variation in *α* can be divided into two distinct regions. In region 1, *α* increases monotonically with increasing d reaching a peak between d = 2–2.4 pC/μm^2^. At higher doses *α* falls rapidly at first followed by a slower decrease with further increase in d. To understand the observed behaviour, the variation in the lower dose regime (region 1) for NiFe/Pt was compared with that of NiFe(10 nm)/Cu(3 nm) bilayer in Fig. 3(c). In both cases *α* increased linearly with increasing d at similar rate of ~0.015 μm^2^/pC, but with a constant higher offset of 0.019 in the case of the NiFe/Pt bilayer. Significant spin-pumping and SOC associated with the Pt at the interface can explain the origin of the shift. There are several studies related to the enhancement of *α* to spin pumping and SOC in the interfacial region, describing the Pt layer as a spin sink that absorbs spin waves from the adjacent FM layer^31^,^32^,^33^. The effect is insignificant in case of NiFe/Cu^33^ which is supported by the observation that for NiFe/Cu *α* approaches 0.015 at d = 0, which is comparable with the damping coefficient of uncapped NiFe. SOC and spin pumping cannot explain the observed linear increase in *α* with dose that is common to both sets of samples. The doping from Ga is less than 1–2% and this is not expected to significantly affect the damping of pure NiFe^25^. However, with ion beam irradiation, NM atoms are displaced into the NiFe causing defects and structural changes that give rise to extrinsic two magnon scattering, which causes an enhancement in *α*^34^. The defects introduced in the NiFe layer increase the width of intermixed region as the irradiation dose increases^24^, resulting in a linear variation of *α* with d. This is supported by earlier literature in case of NiFe/Au bilayers^22^, where it was suggested that the enhanced *α* of NiFe/Au with irradiation was an extrinsic effect attributed to two-magnon scattering. This mechanism has been associated with defects, disorder or misfit symmetry breaking at the interface layer^25^,^35^,^36^. The dominance of two-magnon scattering as the mechanism for enhanced *α* with increasing d in region 1, is also supported by results from studies concerned with interface of an FM layer with Pt^25^,^35^,^37^,^38^,^39^,^40^,^41^. In Fig. 3(b) the variation of *α* with further increase of d shows a monotonic decrease in region 2. The shaded region indicates the dose region where *α* is expected to be maximum according to the trend of experimental data points. Structurally, ion beam irradiation leads predominantly to ballistic intermixing and some insight into the evolution of the NiFe/Pt interface may be gained from previous detailed x-ray structural analysis of FIB irradiated NiFe/Au bilayers^24^,^25^,^40^,^42^ since the process is dominated by momentum transfer and the atomic mass of Pt is close to that of Au. The interface in as-deposited NiFe/Au and NiFe/Pt has some small intrinsic width (~1 nm from XRR measurements) and with increasing irradiation dose the interface width increases linearly due to intermixing of the layers, creating an increasing thickness of compositionally-graded NiFePt alloy between the NiFe and Pt. Previous experimental analysis of the Au capped system showed that a dose of roughly 1 pC/μm^2^ creates a graded alloy interlayer of ~4 nm and sputter loss of more than 1 nm of NM capping layer. Extending this to the NiFe/Pt system, and assuming continued linear intermixing, for a dose above 2 pC/μm^2^ this would lead to the formation of a compositionally graded-alloy that extends through the NiFe layer thickness and the loss of most of the pure NM capping layer^24^,^25^,^34^. Here, the compositionally-graded alloy region forms a continuous magnetic layer with the remaining NiFe part of the film. In addition the loss of pure Pt layer thickness due to sputtering of the Pt-rich surface region leads to a reduction of the SOC and interlayer spin diffusion contributing to the intrinsic *α*. As a result the effective *α* falls.

Further analysis of the variation of *α* with *f* is shown in Fig. 3(d). In the sample with minimum dose (d = 0.3 pC/μm^2^), *α* is independent of *f*, which is a signature of intrinsic damping. For doses of d = 2.0 and 3.3 pC/μm^2^ *α* decreases steadily with *f* with similar slopes indicating an extrinsic contribution to the damping in both the cases. The extrinsic effect is dominant when *H* is weak (lower frequencies). At higher field *f* is higher and the magnetization dynamics are strongly driven by the field itself and hence scattering is suppressed. Hence, the enhancement in *α* with decreasing *f* is a characteristic of two-magnon scattering effect. From the difference in slope in *α* (*f*) between d = 0.3 and 2.0 pC/μm^2^ curves in Fig. 3(d), it is clear that the extrinsic damping develops with intermixing which supports our earlier explanation for linear variation of *α* (d) in region 1 of Fig. 3(b) and the similar slope for d = 2.0 and 3.3 pC/μm^2^ suggests the extrinsic contribution continues in region 2. This is because further irradiation beyond a certain dose does not increase the number of scattering defects in the pure NiFe layer due to formation of a thick interface region.

**Figure 4:**
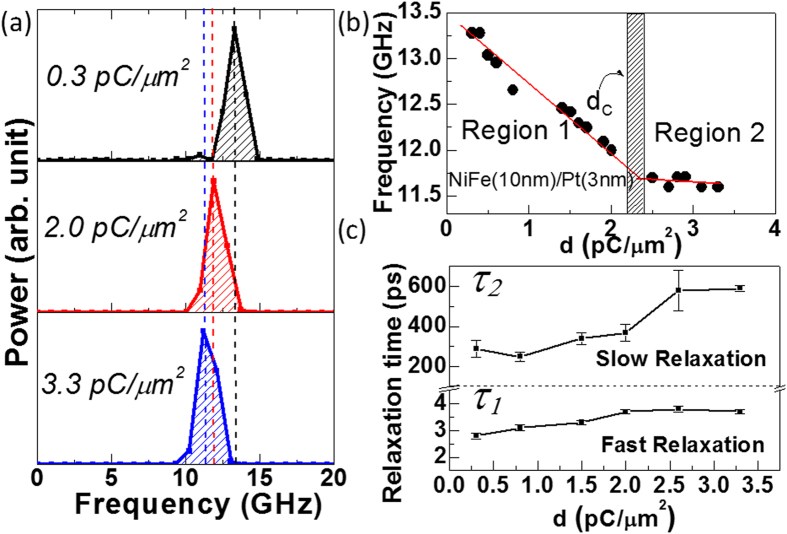
(**a**) FFT power spectra of the TR-MOKE data of ion irradiated NiFe/Pt samples at three different doses at *H* = 1.8 kOe. (**b**) Variation of frequency *f* as a function of dose at *H* = 1.8 kOe. Symbols are the experimental data and solid lines are linear fits. The shaded box represents the transion between two regions. (**c**) Magnetization relaxation times *τ*_*1*_ and *τ*_*2*_ are plotted as a function of dose. Here the symbols are obtained from experimental data while the solid lines are only guides to the eye.

Figure 4(a) shows FFT power spectra of the TR-MOKE data for three different doses. A clear decrease of *f* is observed with increasing dose. The variation of *f* with d is shown in Fig. 4(b). First a large decrease of *f* of 1.3 GHz occurs as d increases from 0.3 to 2.0 pC/μm^2^, while a smaller fall of 0.4 GHz occurs as d increases further from 2.0 to 3.3 pC/μm^2^. The literature^43^ shows a reduction of magnetic moment of Ni thin film with the concentration of Pt atoms. Hence, the decrease of *f* in region 1 is likely to be related to the reduction of moment due to the presence of Pt in the vicinity of Ni and Fe in the intermixed region. In region 2 a NiFe-Pt alloy is established throughout a large fraction of the original bilayer. The precessional frequency is almost constant with increasing dose, because the probability of sputtered Pt atoms to reach the pure NiFe layer by traversing through the thick interface region is small. This effect is in agreement with the structural evolution discussed earlier.

### Spin relaxation times

In Fig. 4(c) we plot the relaxation times 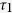 and 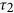 as a function of d. The relaxation times are obtained from the decaying part of the time-resolved Kerr rotation data as discussed earlier in Fig. 1(b). As mentioned earlier that 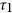 is related to the transfer of electron and spin energy to the lattice, while 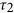 is related to the transfer of lattice energy to the substrate and surroundings. The energy transfer rate depends on the specific heat of lattice (S). In other words 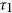 and 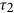 can be expressed in terms of S. Figure 4(c) shows both 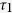 and 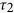 increase with d. Specifically, the change is significant (larger than error) before and after d = 2.0 pC/μm^2^. This is an indirect indication of a change in lattice configuration due to the formation of alloyed layer in region 2.

### Summary

In summary, the controlled modification of dynamic magnetic properties by low dose Ga^+^ ion irradiation in NiFe/Pt bilayer films has been demonstrated. TR-MOKE microscopy was used to study the variation of ultrafast magnetization dynamics including the ultrafast relaxation times, precession frequency and damping as a function of the irradiation dose. These parameters all show significant variation with dose, which has been divided into two regions. A lower dose region (≤2.0 pC/μm^2^); where the precessional frequency falls and the damping increases almost linearly with dose, and a higher dose region (>2.4 pC/μm^2^), where the precessional frequency remains nearly unchanged while the damping decreases slowly. The damping of very low dose irradiated sample shows a large value as compared to uncapped NiFe layer due to the spin-orbit coupling and spin-pumping effects. Low dose irradiation causes dislocation of interface atoms leading towards defects and interface intermixing, which increases two-magnon scattering and the associated damping. With further increase of irradiation dose the interface region expands through the bilayer leading to the formation of a compositionally graded alloy. In addition to that a progressive loss of the Pt layer by sputtering reduces the thickness of this spin-sink region below the spin-diffusion length, which in turn reduces the spin-orbit coupling and spin diffusion process and hence the damping of the system. The two relaxation times which are related to thermal conduction and lattice structural changes also show concurrent variation, confirming the mechanisms as discussed above. Such a controlled variation of damping and precession frequency by low dose local ion irradiation may be important for the development of various spintronic and magnonic devices with patterned static and dynamic magnetic properties.

## Methods

### Fabrication

A bilayer of Ni_81_Fe_19_(10 nm)/Pt(3 nm) was grown by sputtering on a thermally oxidised Si[100] substrate using a ultrahigh vacuum deposition system from a base pressure ~1 × 10^−8^  Torr. Sputter deposition used Ar at a pressure ~1 × 10^−3^ Torr and the deposition rates were 0.01 nm/s and 0.02 nm/s for the NiFe and Pt, respectively. The bilayered films were patterned by electron beam lithography using deposition and lift-off into 30 μm disks and were irradiated with 30 keV Ga^+^ ions at normal incidence using FEI Helios NanoLab 600 FIB system with the focused beam rastered scan over a circular area of ~700 μm^2^. The irradiation dose (d) was varied from 0 to 3.3 pC/μm^2^.

### Measurement

A two-color optical pump-probe setup is used to measure ultrafast magnetization dynamics with spatial and temporal resolutions of ~1μm and 100 fs, respectively. The sample is excited using a second harmonic (λ_B_ = 400 nm, fluence ~17.0 mJ/cm^2^, pulsewidth ~100 fs) of a mode locked Ti-sapphire pulsed laser (Tsunami, Spectra physics, pulsewidth ~80 fs). The fundamental laser beam (λ_R_ = 800 nm, fluence ~2.1 mJ/cm^2^) is used to probe the dynamics passing through a variable time delay. The polar Kerr rotation is detected using a balanced photo diode detector, which completely isolates the Kerr rotation and reflectivity signals. The pump and probe beams are made collinear and are focused on the central part of the sample through a microscope objective with numerical aperture, N.A. = 0.65. At the focal plane of the probe (diameter ~800 nm), i.e., on the sample surface, the pump beam is slightly defocused, and has a larger diameter (~1 μm) than the probe beam, which makes it easier to overlap the pump and probe beams on the sample surface. The probe beam is centred on the pump beam so that slight misalignment during the course of the experiment does not affect the pump-probe signals. A large enough magnetic field is first applied at a small angle ~5° to the planes of the sample for saturating their magnetization. The magnetic field strength is then reduced to the bias field value (*H* = component of bias field in the sample plane), which ensures that the magnetization still remains saturated along the bias field direction. The pump beam was chopped at 2 kHz frequency and a phase sensitive detection of total reflectivity and Kerr rotations were made using lock-in amplifiers. For obtaining the scanning Kerr images the sample is scanned under the focused laser spot by using a piezoelectric scanning stage (x-y-z) with feedback loop for better stability.

## Additional Information

**How to cite this article**: Ganguly, A. *et al.* Tunable Magnetization Dynamics in Interfacially Modified Ni_81_Fe_19_/Pt Bilayer Thin Film Microstructures. *Sci. Rep.* **5**, 17596; doi: 10.1038/srep17596 (2015).
